# The *p*-orbital magnetic topological states on a square lattice

**DOI:** 10.1093/nsr/nwab114

**Published:** 2021-06-28

**Authors:** Jing-Yang You, Bo Gu, Gang Su

**Affiliations:** Kavli Institute for Theoretical Sciences, and CAS Center for Excellence in Topological Quantum Computation, University of Chinese Academy of Sciences, Beijing 100190, China; Department of Physics, Faculty of Science, National University of Singapore, Singapore 117551, Singapore; Kavli Institute for Theoretical Sciences, and CAS Center for Excellence in Topological Quantum Computation, University of Chinese Academy of Sciences, Beijing 100190, China; Physical Science Laboratory, Huairou National Comprehensive Science Center, Beijing 101400, China; Kavli Institute for Theoretical Sciences, and CAS Center for Excellence in Topological Quantum Computation, University of Chinese Academy of Sciences, Beijing 100190, China; Physical Science Laboratory, Huairou National Comprehensive Science Center, Beijing 101400, China; School of Physical Sciences, University of Chinese Academy of Sciences, Beijing 100049, China

**Keywords:** *p*-orbital magnetism, square lattice, topological states

## Abstract

Honeycomb or triangular lattices were extensively studied and thought to be proper platforms for realizing the quantum anomalous Hall effect (QAHE), where magnetism is usually caused by *d* orbitals of transition metals. Here we propose that a square lattice can host three magnetic topological states, including the fully spin-polarized nodal loop semimetal, QAHE and the topologically trivial ferromagnetic semiconductor, in terms of the symmetry and *k* · *p* model analyses that are material independent. A phase diagram is presented. We further show that the above three magnetic topological states can indeed be implemented in the two-dimensional (2D) materials ScLiCl_5_, LiScZ_5_ (Z=Cl, Br) and ScLiBr_5_, respectively. The ferromagnetism in these 2D materials is microscopically revealed from *p* electrons of halogen atoms. This present study opens a door to explore the exotic topological states as well as quantum magnetism from *p*-orbital electrons by means of the material-independent approach.

## Teaser

Based on lattice symmetry and *k* · *p* model analyses, we propose the p-orbital magnetic topological states on square lattice, including topological semimetals, QAHE and ferromagnetic semiconductors, and predict several example materials by first-principles calculations.

## INTRODUCTION

In two-dimensional (2D) systems, the coexistence of magnetism and non-trivial topological states can induce many novel physical phenomena. A typical example is the quantum anomalous Hall effect (QAHE), where the combination of ferromagnetism and topological insulator can generate dissipationless edge states at boundaries [1–6]. The quantized Hall conductivity is carried by the edge states, which are robust against disorders and impurities. Owing to the dissipationless chiral edge states, QAHE would have potential applications in ultralow-power consumption spintronic devices [7]. Thus, the search for materials with QAHE has attracted extensive interest [3,8–12]. Since the seminal work of Haldane [1], the honeycomb lattice is thought to be a proper platform to realize the QAHE, e.g. several ferromagnetic transition metal trihalides with honeycomb lattice were proposed to be candidates for the implementation of QAHE [13–19]. Besides, the experimental observation of QAHE was realized in magnetic atom doped systems [10,20–22], and recently in the few layers of magnetic semiconductor MnBi_2_Te_4_ [23–26] with triangular lattice. In these materials, *d* orbitals of the transition metal play important roles in realizing QAHE. The following two interesting questions then arise. Can the QAHE be realized in other lattices, such as the square lattice? Can the QAHE be obtained in materials with *p* orbitals? The studies of these questions not only give a further understanding of topological states and quantum magnetism, but also offer a new family of materials to search for possible room-temperature QAHE.

In this work, we address these appealing issues by revealing a square lattice with the space group P/4n (No. 85) that can accommodate three different *p*-orbital magnetic topological states, i.e. the fully spin-polarized nodal loop semimetal, QAHE and the ferromagnetic semiconductor. These three quantum states can be obtained by the symmetry and *k* · *p* model analysis, which can be implemented in the 2D materials ScLiCl_5_, LiScZ_5_ (Z=Cl, Br) and ScLiBr_5_. It is shown that the ferromagnetism in these 2D materials is attributed to *p* orbitals. Our findings provide a new mechanism of magnetic topological states from *p*-orbital electrons on square lattices, and also present a novel family of 2D magnetic topological materials with high Chern number.

## THREE MAGNETIC TOPOLOGICAL STATES ON SQUARE LATTICES

**Figure 1.**
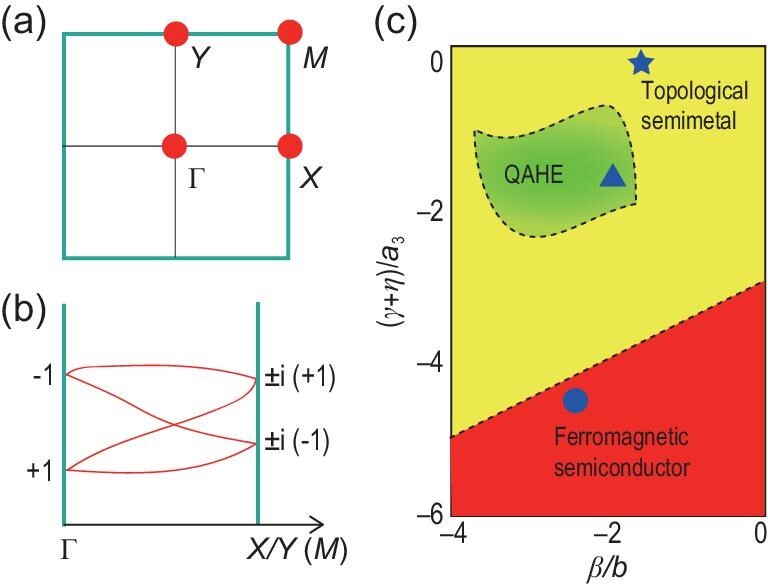
The square lattice with three magnetic topological states. (a) Schematic illustration of the double degeneracy at high-symmetry points (red dots). (b) Schematic depiction of hourglass dispersion along Γ − *X*/*Y* and Γ − *M* high-symmetry lines. The labels indicate the eigenvalues of $\widetilde{M}_z$. (c) Schematic phase diagram with respect to the parameters (γ + η)/*a*_3_ and β/*b*_1_ in (1), where yellow, red and green regions represent topological semimetal, topologically trivial ferromagnetic semiconductor and QAHE states, respectively.

Let us consider a square lattice with the space group of P/4n (No. 85) for *p* orbitals. There are three generators: four-fold rotation symmetry *C*_4_: (*x*, *y*, $z$) → (*y*, −*x*, $z$), roto-inversion symmetry $\tilde{I}$: $(x,y,z)\rightarrow (\frac{1}{2}-x,\frac{1}{2}-y,-z)$ and glide mirror symmetry $\widetilde{M}_z$: $(x,y,z)\rightarrow (\frac{1}{2}+x,\frac{1}{2}+y,-z)$. The symmetry-protected double degeneracy appears at high-symmetry points in the absence of spin-orbit coupling (SOC), as shown in Fig. 1(a). Without SOC, the spin and orbital parts of the electronic wave functions are decoupled, and hence all crystalline symmetries are preserved for each spin channel separately like spinless particles. For the whole Brillouin zone (BZ), the glide mirror $\widetilde{M}_z$ is preserved. The high-symmetry points Γ, *X*, *Y* and *M* are invariant under the combined operation $T\widetilde{M}_z$. We note that $(T\widetilde{M}_z)^2 = T_{110}$, where *T*^2^ = 1 for the spinless case, and $T_{110}=e^{-ik_x-ik_y}$ represents the translation by one unit cell along the [110] direction. At point *X*, we have *k*_*x*_ = π and *k*_*y*_ = 0, while at point *Y*, we have *k*_*x*_ = 0 and *k*_*y*_ = π. Consequently, $(T\widetilde{M}_z)^2=-1$ for points *X* and *Y*. This antiunitary operator thus generates a Kramers-like double degeneracy at points *X* and *Y*. One may note that point *M* is invariant under both *C*_4_ and $\widetilde{I}$. The commutation relation between *C*_4_ and $\widetilde{I}$ is given by $C_4\widetilde{I}=T_{0\overline{1}0}\widetilde{I}C_4$, where $T_{0\overline{1}0}=e^{ik_y}$. At point *M*, we have *k*_*x*_ = π and *k*_*y*_ = π; hence, $T_{0\overline{1}0} = -1$. As a result, for any energy eigenstate |*u*〉 with *C*_4_ eigenvalue *E*_$z$_, it must have a degenerate partner $\widetilde{I}|u\rangle$ with *C*_4_ eigenvalue -*E*_$z$_. This proves that the double degeneracy at *M* is guaranteed by the symmetry. At point Γ, *p*_*x*_ and *p*_*y*_ orbitals should be degenerate, while *p*_$z$_ orbitals are not.

Now we turn to discuss the possible band crossing at high-symmetry lines. Consider the Γ − *X* and Γ − *M* lines, which are invariant under $\widetilde{M}_z$. The Bloch states along Γ − *X* can be chosen as the eigenstates of $\widetilde{M}_z$ with eigenvalues $E_z=\pm e^{-ik_x/2}$. The glide eigenvalues are ±*i* at *X* and ±1 at Γ. Because Γ and *X* are both time-reversal-invariant momenta, a Kramers pair has eigenvalues (+*i*, −*i*) at *X*, and yet it has (+1, +1) or (−1, −1) at Γ. This suggests that the pairs must switch partners when going from *X* to Γ, and the switching leads to the hourglass-type dispersion, as schematically shown in Fig. 1(b). A similar analysis applied for path Γ − *Y* shows that the band crossing is also hourglass type along Γ − *Y*. The Bloch states along Γ − *M* have the eigenstates of $\widetilde{M}_z$ with eigenvalues $E_z=\pm e^{-ik_x/2-ik_y/2}$. At the Γ (0, 0) point, the eigenvalues are (+1, −1), while at the *M* (π, π) point the eigenvalues are (−1, +1). Focusing on the middle of the two bands, they have opposite eigenvalues, and their ordering is inverted between Γ and *M*. As a result, there must be a cross along the Γ − *M* path, and the crossing point is protected by $\widetilde{M}_z$ [Fig. 1(b)]. Because Γ and *M* are both time-reversal-invariant momenta, a Kramers pair with eigenvalues +1 (or −1) is degenerate, leading to the crossing point of hourglass type. The crossing point may trace out a nodal loop centered at Γ.

To characterize the above discussed band crossing, we construct an effective *k* · *p* model for the low-energy band structure on a square lattice. We first consider the case without SOC. The four states at point Γ correspond to two 2D irreducible representations *E*_*u*_ and *E*_*g*_. The model should respect the following symmetries: the four-fold rotation *C*_4$z$_ and the mirror symmetry *M*_$z$_. Expanding up to the *k* quadratic order, we find that the effective Hamiltonian takes the form

$$\begin{eqnarray*}
H_{0}= \left(\begin{array}{cccc}a(k) &\,\,\,\, -ic(k) &\,\,\,\, 0&\,\,\,\, 0 \\
ic(k)) &\,\,\,\, a(k)&\,\,\,\, 0&\,\,\,\, 0\\
0&\,\,\,\, 0&\,\,\,\, b(k)&\,\,\,\, -id(k)\\
0&\,\,\,\, 0&\,\,\,\, id(k)&\,\,\,\, b(k) \end{array}\right),
\end{eqnarray*}$$

where *a*(*k*) = *a*_1_ + *a*_2_*k*^2^, *b*(*k*) = *b*_1_ + *b*_2_*k*^2^, *c*(*k*) = *a*_3_ + *a*_4_*k*^2^, *d*(*k*) = *b*_3_ + *b*_4_*k*^2^, $k^2=k_x^2+k_y^2$ and the parameters *a*_*i*_ and *b*_*i*_ (*i* = 1, 2, 3, 4) are real. Considering a fully spin-polarized ferromagnet, the inclusion of SOC gives an additional contribution to the above Hamiltonian, which can be treated as a perturbation due to the relatively weak SOC strength. We find that the SOC term up to the leading order takes the form

$$\begin{equation*}
H_{\rm SOC}= \left(\begin{array}{cccc}\alpha &\,\,\, \gamma -\gamma i &\,\,\, 0&\,\,\, 0 \\
\gamma +\gamma i &\,\,\, \alpha &\,\,\, 0&\,\,\, 0\\
0&\,\,\, 0&\,\,\, \beta &\,\,\, \eta -\eta i\\
0&\,\,\, 0&\,\,\, \eta +\eta i&\,\,\, \beta \end{array}\right),
\end{equation*}$$

with real parameters α, β, γ and η. Thus, the total Hamiltonian reads

(1) \begin{equation*} H=H_0+H_{\rm SOC}.\end{equation*}

Because of the two-fold degeneracy at point Γ in the presence of SOC, we can obtain *a*_1_ = *b*_1_ and *a*_3_ = *b*_3_. If we fix β = −α, a schematic phase diagram of the ratio of (γ + η)/*a*_3_ as a function of β/*b*_1_ can be drawn, as shown in Fig. 1(c). Obviously, the SOC term will break the degeneracies at point Γ. It may also affect the degeneracy of the nodal loop, i.e. the nodal loop can be preserved with its shape and size changed slightly or the nodal loop vanishes with a band gap opened, and the system becomes QAH insulators or topologically trivial ferromagnetic semiconductors. From Fig. 1(c), one may note that a topologically trivial ferromagnetic semiconductor appears in the region where the SOC parameters β and γ + η have relatively large absolute values, and topologically non-trivial states including the nodal loop semimetal and topological insulator (QAHE) depend on the relationship and competition between β and γ + η. For the QAHE, the band inversion occurs.

## MAGNETIC TOPOLOGICAL MATERIALS WITH SQUARE LATTICES

**Figure 2.**
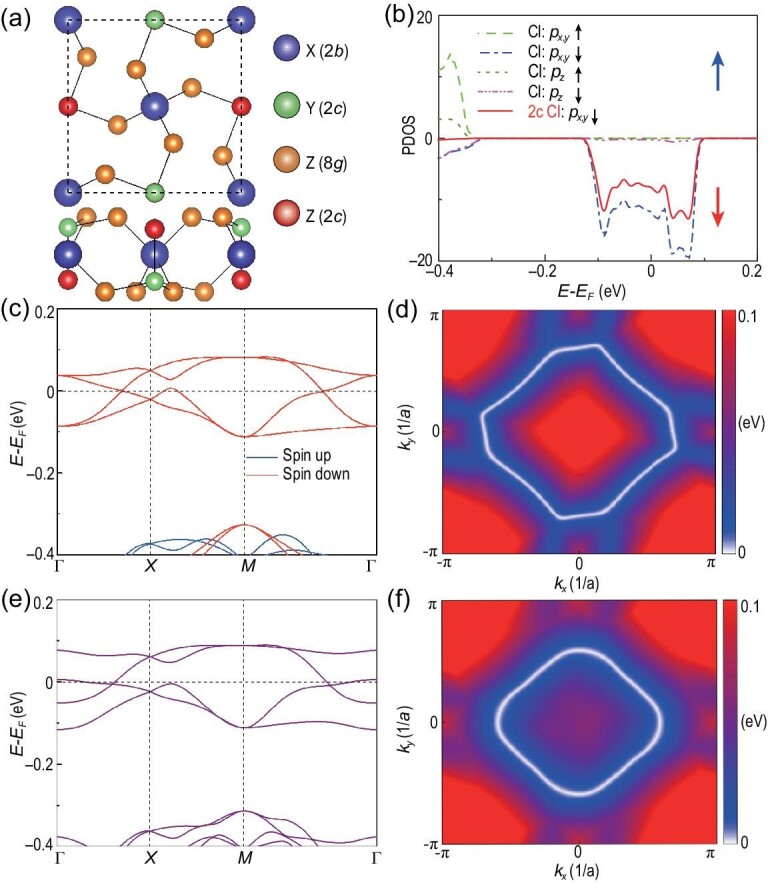
Fully spin-polarized nodal loop semimetal ScLiCl_5_ monolayer. (a) Top and side views of 2D materials XYZ_5_, where X atoms occupy the Wyckoff position 2*b*(0; 0; 0.5) colored in blue, Y atoms occupy the Wyckoff position 2*c*(0.5; 0; 0.56383) colored in green and Z atoms occupy the Wyckoff positions 8*g*(0.20444, 0.11338, 0.59774) (orange) and 2*c*(0.5, 0, 0.40678) (red). (b) Partial density of states, (c) band structure and (d) the Weyl loop obtained from density functional theory (DFT) calculations in the absence of SOC for the ScLiCl_5_ monolayer. (e) Electronic band structure and (f) the Weyl loop obtained from DFT calculations of ScLiCl_5_ with SOC.

We now present several 2D material examples to implement the above different topological states, i.e. topological semimetal, topologically trivial ferromagnetic semiconductor and QAHE states, as indicated in Fig. 1(c). These 2D materials with the formula unit XYZ_5_ possess a square lattice with the space group of P/4n, where X atoms occupy the Wyckoff position 2*b*(0; 0; 0.5), Y atoms occupy the Wyckoff position 2*c*(0.5; 0; 0.56383) and Z atoms occupy the Wyckoff positions 8*g*(0.20444, 0.11338 and 0.59774) and 2*c*(0.5, 0 and 0.40678) as shown in Fig. 2(a). It is interesting to mention that several bulk materials with similar structures have been synthesized and extensively studied in the last decades [27–33].

### Fully spin-polarized nodal loop semimetal in ScLiCl_5_

The structure of the ScLiCl_5_ monolayer with a square lattice is depicted in Fig. 2(a). Each primitive cell contains two formula units of XYZ_5_. To confirm the stability of the ScLiCl_5_ monolayer, its phonon spectra have been calculated. There is no imaginary frequency mode in the whole Brillouin zone, indicating that this monolayer is dynamically stable. The structural stability of the ScLiCl_5_ monolayer is also examined in terms of the formation energy. The obtained negative values of the formation energy (the energy difference between XYZ_5_ and X, Y crystals, 5/2 Z_2_ molecule) for XYZ_5_ monolayers are indicative of an exothermic reaction. Moreover, the thermal stability of the ScLiCl_5_ monolayer is tested using molecular dynamics simulation by considering a 3 × 3 × 1 supercell of ScLiCl_5_ with 126 atoms. After being heated at 300 K for 6 ps with a time step of 3 fs, no structural changes occur, indicating that this monolayer is also thermodynamically stable. More details can be found in the online supplementary material.

The optimized lattice constant of the ScLiCl_5_ monolayer is *a*_0_ = 7.9372 Å. By a spin-polarized calculation, we note that the total spin magnetic moment carried by ScLiCl_5_ is about 1.53 μ_*B*_ per unit cell, which is mainly attributed to the two Cl atoms at Wyckoff position 2*c* [colored red in Fig. 2(a)], whose spin and orbital moments are of about 0.50 and 0.16 μ_*B*_ per atom, respectively. The ferromagnetism mainly originates from the *p* orbitals of Cl atoms, whereas the spin magnetic moment on the Sc atom is calculated to be zero. It is well interpreted that, for ScLiCl_5_, because Li and Sc have one and three valence electrons, respectively, Cl atoms possess unpaired electrons and thus should carry a non-zero spin magnetic moment. To determine the magnetic ground state, we compared the total energies between ferromagnetic (FM), antiferromagnetic (AFM) and non-magnetic (NM) states. The FM state is found to be more stable than the AFM and NM states.

The partial density of states (PDOS) and electronic band structure in the absence of SOC are shown in Fig. 2(b) and (c), respectively. One observes that the material is a half-metal, with only one spin channel (spin down) being metallic and another spin channel (spin up) being insulating. From the projected density of states as displayed in Fig. 2(b), one may see that the states around the Fermi energy are fully polarized in the spin-down channel, while the spin-up channel has a large gap. In addition, the low-energy states are dominated by the *p*_*x*, *y*_ orbitals of the Cl atoms at Wyckoff position 2*c*. From Fig. 2(c), we find two features of the band structure. One is the double degeneracy at high-symmetry points Γ, *X* and *M*, and another is the linear band-crossing points appearing on the paths Γ − *X* and Γ − *M*. These crossing points are not isolated, and form a nodal loop around the Γ point as shown in Fig. 2(d).

In the presence of SOC, magnetic anisotropy should be considered. In order to determine the easy axis of magnetization, we pin down the magnetization direction for FM configurations. By comparing the energies of different magnetization directions, we find that the out-of-plane direction is energetically preferred over the in-plane directions and along that are isotropic. We have also estimated the Curie temperature *T*_*C*_ for the FM state by using the Monte Carlo simulation based on an effective Hamiltonian

(2) \begin{equation*} H_{spin}=\sum _{\langle i,j \rangle }J_1S_i^zS_j^z+\sum _{\langle \langle i,j \rangle \rangle }J_2S_i^zS_j^z, \end{equation*}

where the spin vectors are normalized, the superscripts *i* and *j* label the 2*c* Cl sites, 〈*i*, *j*〉 and 〈〈*i*, *j*〉〉 indicate nearest-neighboring and next-nearest-neighboring sites, respectively, and *J*_1_ and *J*_2_ are the corresponding FM exchange integrals. The values of *J*_1_ and *J*_2_ extracted from DFT calculations are −4.572 and −0.161 meV, respectively. The calculated Curie temperature for monolayer ScLiCl_5_ is about 123 K.

The electronic band structure with SOC for the ScLiCl_5_ monolayer is shown in Fig. 2(e). Note that the SOC only breaks the degeneracy at point Γ, but keeps the degeneracies at points *X*(*Y*) and *M*, which are protected by symmetry. The nodal loop is also preserved with SOC in monolayer ScLiCl_5_, as shown in Fig. 2(f). Thus, the ScLiCl_5_ monolayer exhibits a fully spin-polarized nodal loop semimetal (we could call it the nodal loop half-semimetal). By fitting the two bands near the Fermi level, we can obtain the parameters for ScLiCl_5_: β/*a*_1_ = −1.4 and (γ + η)/*a*_3_ = 0. Thus, ScLiCl_5_ locates in the region of the topological semimetal marked by a star in Fig. 1(c).

### Ferromagnetic semiconductor in ScLiBr_5_

**Figure 3.**
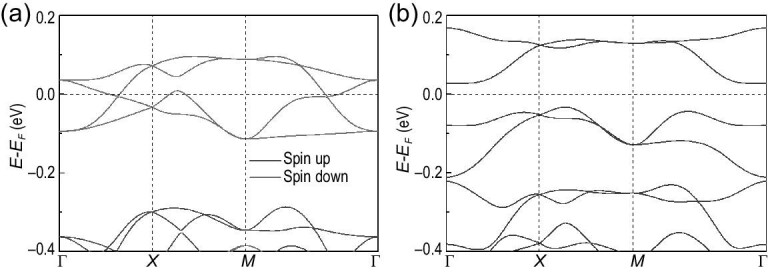
Ferromagnetic semiconductor ScLiBr_5_ monolayer. The electronic band structure (a) without SOC and (b) with SOC.

The ScLiBr_5_ monolayer shares the same structure as ScLiCl_5_, except a larger lattice constant *a* = 8.4175 Å. The stability of monolayer ScLiBr_5_ is also checked by its phonon spectra, molecular dynamics and formation energy, indicating it is feasible in experiment (see the online supplementary material). The spin-polarized calculation shows that the total spin magnetic moment of ScLiBr_5_ is about 1.43 μ_*B*_ per unit cell, and the two Br atoms at Wyckoff position 2*c* possess spin and orbital magnetic moments of about 0.46 and 0.34 μ_*B*_ per atom, respectively, whereas spin and orbital magnetic moments on other atoms are negligible. By comparing the total energies between the FM, AFM and NM states, the FM state is found to be more stable than the antiferromagnetic and non-magnetic states. The ScLiBr_5_ monolayer has a similar band structure in the presence of SOC, as shown in Fig. 3(a). In this case, the ScLiBr_5_ monolayer possesses a ferromagnetic ground state with out-of-plane magnetization, and the Curie temperature was estimated to be 67 K by Monte Carlo simulation based on (2) with *J*_1_ = −1.990 meV and *J*_2_ = −0.455 meV. The band gap of about 60.2 meV is opened by SOC for monolayer ScLiBr_5_, and it turns into a topologically trivial ferromagnetic semiconductor state with a zero Chern number. By fitting the two bands near the Fermi level, we can obtain the parameters for ScLiBr_5_: β/*a*_1_ = −2.3 and (γ + η)/*a*_3_ = −4.6, which locates in the region of the ferromagnetic semiconductor marked by a dot in Fig. 1(c).

### QAHE in LiScX_5_ (X=Cl, Br)

The LiScX_5_ (X=Cl, Br) monolayer can be obtained by exchanging the positions of the Li and Sc atoms in the structures of ScLiCl_5_ and ScLiBr_5_ [Fig. 2(a)]. We take LiScCl_5_ as a prototypal example because LiScBr_5_ shares very similar features. The optimized lattice constant of the LiScCl_5_ monolayer is *a*_0_ = 8.0048 Å. The stability of monolayer LiScCl_5_ is also checked by its phonon spectra, molecular dynamics and formation energy (see the online supplementary material), indicating it is feasible in experiment. By comparing the formation energy, we find that ScLiCl_5_ (−1.52 eV) is a little more stable than LiScCl_5_ (−1.51 eV). By a spin-polarized calculation, we find that the spin magnetic moment carried by LiScCl_5_ is about 1.54 μ_*B*_ per cell shared by all Cl atoms, while the Cl atoms at Wyckoff position 2*c* have orbital moment (0.03 μ_*B*_) much larger than that (0.01 μ_*B*_) for the Cl atoms at Wyckoff position 8*g*. Our calculated results show that the FM state is more stable than the antiferromagnetic and non-magnetic states.

**Figure 4.**
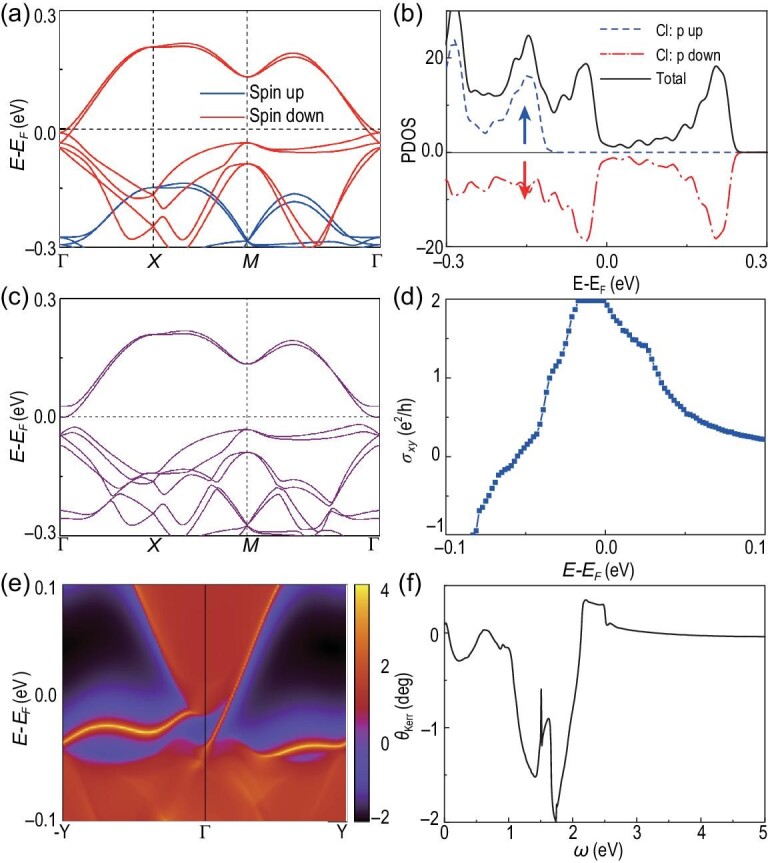
QAHE in the LiScCl_5_ monolayer. (a) Band structure in the absence of SOC and (b) partial density of states. (c) The band structure, (d) anomalous Hall conductivity and (e) projected spectrum on the (100) surface (line for 2D) with SOC. (f) The Kerr angle θ_Kerr_ as a function of photon energy ω.

The electronic band structure and density of states for monolayer LiScCl_5_ in the absence of SOC are shown in Fig. 4(a) and (b), respectively. It can be seen that monolayer LiScCl_5_ holds a similar band structure as ScLiCl_5_ in the presence of SOC except that the band crossing points are closer to the Γ point. From the projected density of states as displayed in Fig. 4(b), we may observe that the states around the Fermi energy are fully polarized in the spin-down channel, while the spin-up channel has a large gap. The low-energy states are dominated by the *p* orbitals of all Cl atoms.

In the presence of SOC, magnetic anisotropy should be considered. By comparing the energies of different magnetization directions, we uncover that the out-of-plane direction is energetically preferred over the in-plane directions, and the energy of out-of-plane magnetization is 1.10 meV lower than that of in-plane magnetization. The Curie temperature is estimated to be 28 K. After tuning on SOC, a gap of about 24.7 meV is opened, as shown in Fig. 4(c). The topologically non-trivial band structure of the LiScCl_5_ monolayer is characterized by a non-zero Chern number *C* = 2 with a quantized charge Hall plateau 2e^2^/h and two gapless chiral edge states connecting the valence and conduction bands, as shown in Fig. 4(d) and (e), respectively. In addition to the QAHE with high Chern number, the magneto-optical Kerr effect, being a kind of non-contact (non-damaging) optical technique, is a powerful tool for measuring the magnetism in 2D materials [11,34]. It can be seen that a large Kerr angle θ_Kerr_ is obtained for the LiScCl_5_ monolayer, particularly for photon energies ω near 1.7 eV, as shown in Fig. 4(f). The maximal Kerr angle for the LiScCl_5_ monolayer is an order of magnitude larger than that for the CrGeTe_3_ monolayer [34], and about 3 times larger than that for bulk Fe [35]. By fitting the two bands near the Fermi level, we can obtain the parameters for LiScCl_5_: β/*a*_1_ = −2.0 and (γ + η)/*a*_3_ = −1.9, which locates in the region of the QAHE marked by a triangle in Fig. 1(c). The high-Chern-number QAHE with a large band gap of about 113 meV can be also implemented in monolayer LiScBr_5_ (see the online supplementary material).

## MAGNETIC SINGLE-ION ANISOTROPY

We take ScLiCl_5_ as an example to discuss the microscopic mechanism of the out-of-plane magnetization and large magnetic anisotropy.

According to second-order perturbation theory, magnetic anisotropy from single-ion anisotropy (SIA) can be described as [36,37]

(3) \begin{equation*} E_{\rm SIA}=\lambda ^2\sum _{o,u}\frac{|\langle \psi _u|L_z|\psi _o\rangle |^2-|\langle \psi _u|L_x|\psi _o\rangle |^2}{\epsilon _u-\epsilon _o}, \end{equation*}

where λ is the SOC constant, *L*_$z$/*x*_ represent the angular momentum operators, and ε_*u*_ and ε_*o*_ are the unoccupied and occupied energies, respectively. A positive value of *E*_SIA_ indicates the out-of-plane magnetization, and the in-plane magnetization otherwise. Equation (3) means that the orbitals near the Fermi energy mainly contribute to the magnetic anisotropy energy (MAE). By calculating the differences in matrix elements squared between two directions of the magnetization for *p* orbitals according to (3), as shown in Table 1, we note that the contributions to MAE from the same spins and from opposite spins between occupied (|ψ_*o*_〉) and unoccupied (|ψ_*u*_〉) states have opposite signs. Positive and negative matrix elements prefer the out-of-plane magnetization and in-plane magnetization, respectively. In our systems, the states near the Fermi energy are mainly contributed by the same spin (spin down) of *p*_*x*, *y*_ orbitals, which should prefer an out-of-plane magnetization.

**Table 1.** The differences in matrix elements squared between two directions of the magnetization (|〈*o*^−^|*L*_$z$_|*u*^−^〉|^2^ − |〈*o*^−^|*L*_*x*_|*u*^−^〉|^2^) and (|〈*o*^+^|*L*_$z$_|*u*^−^〉|^2^ − |〈*o*^+^|*L*_*x*_|*u*^−^〉|^2^) in (3), where *o* and *u* are occupied and unoccupied orbitals, and + and − are majority and minority spin states, respectively.

	*p*_*x*, +_	*p*_*y*, +_	*p*_ $z$ , +_	*p*_*x*, −_	*p*_*y*, −_	*p*_ $z$ , −_
*p*_*x*, +_	0	1	0	0	−1	0
*p*_*y*, +_	1	0	−1	−1	0	1
*p*_ $z$ , +_	0	−1	0	0	1	0
*p*_*x*, −_	0	−1	0	0	1	0
*p*_*y*, −_	−1	0	1	1	0	−1
*p*_ $z$ , −_	0	1	0	0	−1	0

To confirm the above observation, the orbital-resolved *E*_SIA_ was calculated for the ScLiCl_5_ monolayer as listed in Table 2. It is seen that the Li and Sc atoms as well as the Cl atoms at Wyckoff position 8*g* make no contribution to *E*_SIA_, while the main contribution comes from the Cl atoms at Wyckoff position 2*c*, as revealed in Table 2. The value of the (*p*_*x*_, *p*_*y*_) matrix element is dominated and positive, indicating an out-of-plane magnetization, which is consistent with the above analysis. For monolayers ScLiBr_5_ and LiScX_5_ (X=Cl, Br), the same analysis applies and we find that they all prefer the out-of-plane magnetization, which is consistent with our DFT results.

**Table 2.** Orbital-resolved magnetic single-ion anisotropic energy *E*_SIA_ of Cl atoms in the fully spin-polarized nodal loop semimetal ScLiCl_5_, where the dominated *E*_SIA_ comes from the (*p*_*x*_, *p*_*y*_) [or (*p*_*y*_, *p*_*x*_)] matrix element of the Cl atoms at Wyckoff position 2*c*.

		(*p*_*x*_, *p*_*y*_)	(*p*_*x*_, *p*_$z$_)	(*p*_*y*_, *p*_$z$_)
Cl	2*c*	3.08	0.10	−0.44
	8*g*	−0.05	0.00	−0.01

The atomic SOC is calculated as $H_{\rm SOC} = \lambda \mathbf {S}\cdot \mathbf {L}$, where λ related to the atomic number is the coefficient of SOC and $\mathbf {S}$ and $\mathbf {L}$ represent the spin and orbital angular momentum operators, respectively. Although λ is small for the Cl atom (∼331 cm^−1^)[39], its orbital moment is large (∼0.16 μ_*B*_), as shown in Table 3, while in many *d*-orbital magnetic materials though their λ is much larger than that of the Cl atom, their orbital magnetic moments are quenched, and thus in our compounds, the atomic SOC of *p* orbitals is large and opens a gap of tens of meV, which is comparable with and even larger than many *d*-orbital magnetic materials. For example, for monolayer CrGeTe_3_, its λ is 740 cm^−1^[39], while its orbital moment is quenched (∼0.004 μ_*B*_)[38], leading to a small SOC. Because of the large SOC in our compounds, the obtained single-ion anisotropy is large.

**Table 3.** The spin 〈*S*〉 and orbital 〈*O*〉 moments (in μ_*B*_) of Z (or Cr) atoms, MAE (in meV) per formula unit between the out-of-plane and in-plane FM configurations and the Curie temperature T_*c*_ (in kelvins) for XYZ_5_ compounds as well as CrGeTe_3_ [38] for comparison.

	Wyckoff				
Monolayer	position	〈*S*〉	〈*O*〉	MAE	T_*c*_
ScLiCl_5_	2*c*	0.497	0.159	3.512	123
	8*g*	0.066	0.002		
ScLiBr_5_	2*c*	0.460	0.337	7.919	67
	8*g*	0.063	0.002		
LiScCl_5_	2*c*	0.136	0.028	0.504	28
	8*g*	0.162	0.006		
LiScBr_5_	2*c*	0.166	0.069	5.418	20
	8*g*	0.142	0.020		
CrGeTe_3_	—	3.614	0.004	1.850	19

From Table 3, we note that the estimated Curie temperature for our compounds is higher than that of CrGeTe_3_, especially for ScLiCl_5_ and ScLiBr_5_. Moreover, for ScLiCl_5_, ScLiBr_5_ and LiScBr_5_, their magnetic anisotropies are much higher than CrGeTe_3_ because the former has larger orbital magnetic momenta. Thus, the *p*-orbital magnetism in our compounds is reliable and stable.

## CONCLUSION

In this work, we propose the *p*-orbital topological magnetic states on a square lattice with space group P/4n by means of the symmetries and *k* · *p* model analyses that are material independent. Three currently interested topological states, including topological semimetal, QAHE and the topologically trivial ferromagnetic semiconductor, can be obtained on the square lattice, depending on the interplay between different SOC parameters. A phase diagram is presented. As examples, we show that the above three different topological states can indeed be implemented in the 2D materials ScLiCl_5_, ScLiBr_5_ and LiScCl_5_ (or LiScBr_5_), respectively. Furthermore, the ferromagnetism of these 2D ferromagnets is unveiled from the *p* orbitals of halogen elements, and the microscopic origin of ferromagnetism from *p* electrons is elaborated. This present study opens a door to explore not only exotic topological states (e.g. nodal loop half-semimetal), but also the quantum magnetism from *p*-orbital electrons in terms of the model and material-independent analyses.

## Supplementary Material

nwab114_Supplemental_File
