# Supplementary material for: The p-orbital magnetic topological states on a square lattice
Source: Natl Sci Rev. 2021 Jun 28;9(4):nwab114. doi: 10.1093/nsr/nwab114 (PMC9037132; doi:10.1093/nsr/nwab114)
Supplement: nwab114_Supplemental_File [file nwab114_supplemental_file.pdf]

Supplemental materials for "P-orbital magnetic topological states on square lattice"

Jing-Yang You,[1,2] Bo Gu,[1,3,*] and Gang Su[1,3,4,†]

[1] *Kavli Institute for Theoretical Sciences, and CAS Center for Excellence in Topological Quantum Computation, University of Chinese Academy of Sciences, Beijing 100190, China*

[2] *Department of Physics, Faculty of Science, National University of Singapore, 117551, Singapore*

[3] *Physical Science Laboratory, Huairou National Comprehensive Science Center, Beijing 101400, China*

[4] *School of Physical Sciences, University of Chinese Academy of Sciences, Beijing 100049, China*

In this Supplemental Material we provide additional results for: calculation method, crystal structure and stability of monolayers $ScLiZ_5$ and $LiScZ_5$ (Z=Cl, Br), and electronic and topological properties of monolayer $LiScBr_5$.

CALCULATION METHOD

Our first-principles calculations were based on the density-functional theory (DFT) as implemented in the Vienna *ab initio* simulation package (VASP) [1], using the projector augmented wave method [2]. The generalized gradient approximation with Perdew-Burke-Ernzerhof [3] realization was adopted for the exchange-correlation functional. The plane-wave cutoff energy was set to 550 eV. The Monkhorst-Pack k-point mesh [4] of size 13×13×1 was used for the BZ sampling. The structure relaxation considering both the atomic positions and lattice vectors was performed by the conjugate gradient (CG) scheme until the maximum force on each atom was less than 0.0001 eV/Å, and the total energy was converged to 10^{-8} eV with Gaussian smearing method. To avoid unnecessary interactions between the monolayer and its periodic images, the vacuum layer is set to 20 Å. The phonon frequencies were calculated using a finite displacement approach as implemented in the PHONOPY code [5], in which a 3×3×1 supercell and a displacement of 0.01 Å from the equilibrium atomic positions are employed. The surface spectrum was calculated by using the Wannier functions and the iterative Green's function method [6–9].

CRYSTAL STRUCTURE OF MONOLAYERS SCLIZ$_5$ AND LISCZ$_5$ (X=CL, BR)

The crystal structures of monolayers $ScLiCl_5$, $ScLiBr_5$, and $LiScCl_5$ (or $LiScBr_5$) are given in Fig. S1.

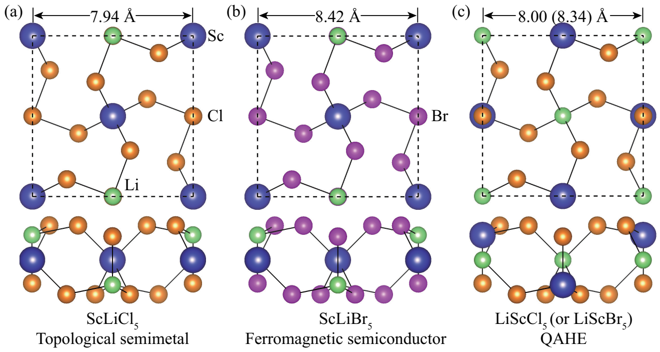

FIG. S1. Crystal structures of (a) 2D magnetic topological semimetal $ScLiCl_5$, (b) ferromagnetic semiconductor $ScLiBr_5$, and (c) magnetic topological insulator $LiScCl_5$ (or $LiScBr_5$).

STABILITY OF MONOLAYERS SCLIZ$_5$ AND LISCZ$_5$ (Z=CL, BR)

The stability of monolayer $ScLiZ_5$ and $LiScZ_5$ (Z=Cl, Br) is checked by their phonon spectra, molecular dynamics and formation energy, indicating they are all feasible in experiment, as shown in Figs. S2, S3 and Table S1.

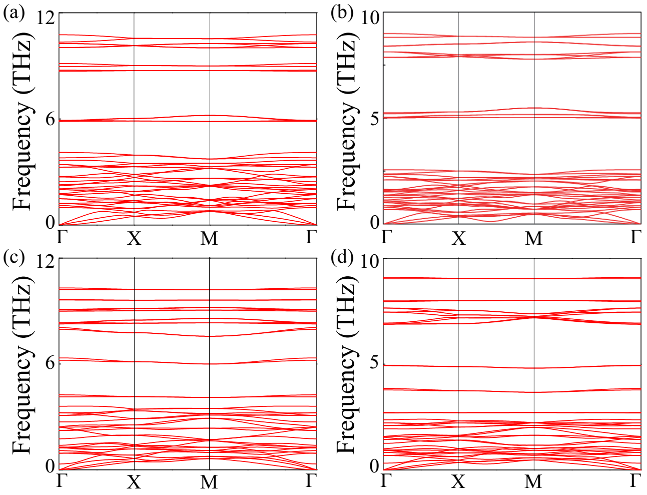

FIG. S2. Phonon spectra of monolayers (a) $ScLiCl_5$, (b) $ScLiBr_5$, (c) $LiScCl_5$ and (d) $LiScBr_5$.

ELECTRONIC AND TOPOLOGICAL PROPERTIES OF MONOLAYER LISCBR$_5$

The monolayer $LiScBr_5$ possesses the same electronic and topological properties as monolayer $LiScCl_5$. The high-Chern-number (C=2) QAHE with a large band gap

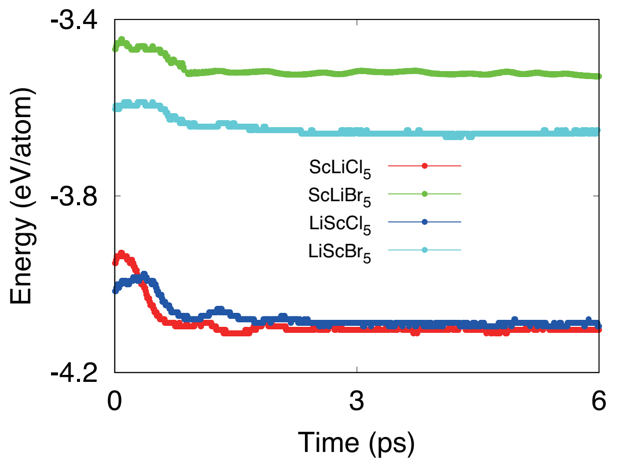

FIG. S3. Molecular dynamics simulation for monolayers $ScLiZ_5$ and $LiScZ_5$ (Z=Cl, Br).

TABLE S1. Formation energy per atom (E_f) of monolayers $ScLiZ_5$ and $LiScZ_5$ (Z=Cl, Br). The formation energy of XYZ_5 monolayers is calculated by E_f = [E(XYZ_5)-E(X)-E(Y)-5/2E(Z_2)]/7, where X, Y and Z_2 are the common elementary substances, i.e fcc bulk of Li and Sc, and Cl_2 (or Br_2) molecules, respectively.

monolayer	$ScLiCl_5$	$ScLiBr_5$	$LiScCl_5$	$LiScBr_5$
E_f (eV)	-1.52	-1.10	-1.51	-1.16

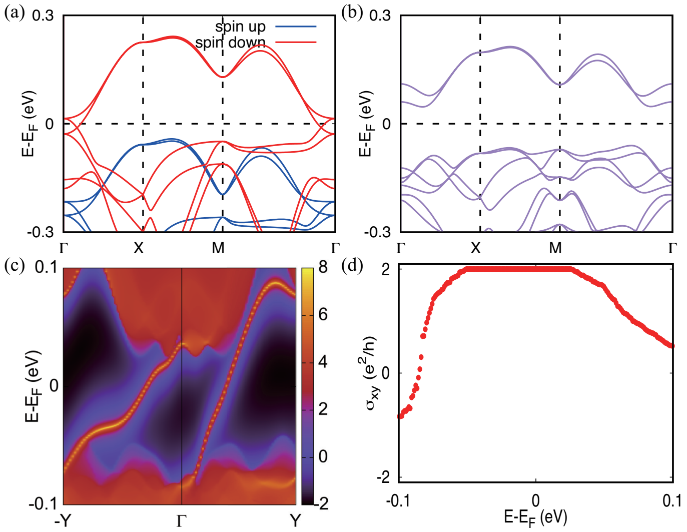

FIG. S4. The band structures of monolayer $LiScBr_5$ (a) without SOC and (b) with SOC. (c) Surface states and (d) anomalous Hall conductivity.

of about 113 meV can be realized in monolayer $LiScBr_5$ as shown in Fig. S4.

* gubo@ucas.ac.cn
† gsu@ucas.ac.cn

[1] G. Kresse and J. Furthmüller, Phys. Rev. B **54**, 11169 (1996).
[2] P. E. Blöchl, Phys. Rev. B **50**, 17953 (1994).
[3] J. P. Perdew, K. Burke, and M. Ernzerhof, Phys. Rev. Lett. **77**, 3865 (1996).
[4] H. J. Monkhorst and J. D. Pack, Phys. Rev. B **13**, 5188 (1976).
[5] A. Togo and I. Tanaka, Scr. Mater. **108**, 1 (2015).
[6] N. Marzari and D. Vanderbilt, Phys. Rev. B **56**, 12847 (1997).
[7] I. Souza, N. Marzari, and D. Vanderbilt, Phys. Rev. B **65**, 035109 (2001).
[8] Q. Wu, S. Zhang, H.-F. Song, M. Troyer, and A. A. Soluyanov, Comput. Phys. Commun. **224**, 405 (2018).
[9] M. P. L. Sancho, J. M. L. Sancho, J. M. L. Sancho, and J. Rubio, J. Phys. F: Met. Phys. **15**, 851 (1985).
[10] U. Wolff, Phys. Rev. Lett. **62**, 361 (1989).
